# Finding Home: Landmark Ambiguity in Human Navigation

**DOI:** 10.3389/fnbeh.2017.00132

**Published:** 2017-07-18

**Authors:** Simon Jetzschke, Marc O. Ernst, Julia Froehlich, Norbert Boeddeker

**Affiliations:** ^1^Department of Biology, Cognitive Neuroscience, Bielefeld University Bielefeld, Germany; ^2^Cognitive Interaction Technology–Cluster of Excellence, Bielefeld University Bielefeld, Germany; ^3^Appl. Cognitive Psychology, Faculty for Computer Science, Engineering, and Psychology, Ulm University Ulm, Germany

**Keywords:** human landmark navigation, multisensory cue integration, landmark integration breakdown, virtual reality, landmark reliabilities, probabilistic navigation model

## Abstract

Memories of places often include landmark cues, i.e., information provided by the spatial arrangement of distinct objects with respect to the target location. To study how humans combine landmark information for navigation, we conducted two experiments: To this end, participants were either provided with auditory landmarks while walking in a large sports hall or with visual landmarks while walking on a virtual-reality treadmill setup. We found that participants cannot reliably locate their home position due to ambiguities in the spatial arrangement when only one or two uniform landmarks provide cues with respect to the target. With three visual landmarks that look alike, the task is solved without ambiguity, while audio landmarks need to play three unique sounds for a similar performance. This reduction in ambiguity through integration of landmark information from 1, 2, and 3 landmarks is well modeled using a probabilistic approach based on maximum likelihood estimation. Unlike any deterministic model of human navigation (based e.g., on distance or angle information), this probabilistic model predicted both the precision and accuracy of the human homing performance. To further examine how landmark cues are integrated we introduced systematic conflicts in the visual landmark configuration between training of the home position and tests of the homing performance. The participants integrated the spatial information from each landmark near-optimally to reduce spatial variability. When the conflict becomes big, this integration breaks down and precision is sacrificed for accuracy. That is, participants return again closer to the home position, because they start ignoring the deviant third landmark. Relying on two instead of three landmarks, however, goes along with responses that are scattered over a larger area, thus leading to higher variability. To model the breakdown of integration with increasing conflict, the probabilistic model based on a simple Gaussian distribution used for Experiment 1 needed a slide extension in from of a mixture of Gaussians. All parameters for the Mixture Model were fixed based on the homing performance in the baseline condition which contained a single landmark. from the 1-Landmark Condition. This way we found that the Mixture Model could predict the integration performance and its breakdown with no additional free parameters. Overall these data suggest that humans use similar optimal probabilistic strategies in visual and auditory navigation, integrating landmark information to improve homing precision and balance homing precision with homing accuracy.

## Introduction

Humans often combine different types of information to find back to a goal location. The proprioceptive, kinaesthetic, and vestibular senses generate information about body posture and movement (Marlinsky, 1999; Mittelstaedt and Mittelstaedt, 2001; Kearns et al., 2002; Angelaki and Cullen, 2008; Souman et al., 2009; Fetsch et al., 2010). A strategy that is used by many animals to find back to a starting position involves integrating these cues for different parts of a journey into a vector pointing home (Etienne and Jeffery, 2004). This mechanism is referred to as *path integration*. Path integration, however, is only effective for backtracking to a previously visited place and depends on potentially noisy (imprecise) and biased (inaccurate) cues (Cheung and Vickerstaff, 2010; Jetzschke et al., 2016). Therefore, many animals, including humans, memorize external reference points for navigation, such as landmarks (Cartwright and Collett, 1987; Gillner and Mallot, 1998; Wehner, 2003; Nardini et al., 2008; Dittmar et al., 2014; Zhao and Warren, 2015). It has been proposed that honeybees and ants use visual snapshots when solving homing tasks. Using this mechanism, the current input is matched to a previously acquired template (Cartwright and Collett, 1987), which can contain information about the spatial configuration of all available landmarks (Waller et al., 2000), about patterns in the skyline (Graham and Cheng, 2009; Philippides et al., 2011), or about the fractional position of mass of a visual pattern (Lent et al., 2013), to name just a few possible sources of information. That is, natural environments can be highly cluttered with landmark cues. It is still largely unclear whether navigating animals explicitly identify and extract specific landmark objects or if they memorize the whole surrounding scenery from the home position, which they then use for visual matching (Gillner et al., 2008; Basten and Mallot, 2010; Philippides et al., 2011; Baddeley et al., 2012; Zeil, 2012; Stürzl et al., 2015). In this context it is also unclear how the different sources of information are combined or whether they are all equally important. Certainly distinct visual shapes which provide precise and accurate spatial information are no doubt important (see Collett et al., 2013 for a review on visual guidance and memory), but is there a weighing of information based on how informative the sources of information are. Framed in terms of spatial constraints the different sources of information only inform about the existence of and the relationship to a certain point in space and all sources are equally important. Additionally, any deterministic model would make no prediction on the precision of navigation performance. That is, they do not probabilistically inform about the precision and thus the importance of the information that a landmark or another spatial cue might provide (cf. Weiss, Simoncelli, Adelson, Nat. Neuro. 2002 for a similar discussion in the visual motion domain). Would that really be a reasonable integration strategy?

When landmark cues are unreliable (imprecise) and ambiguous (inaccurate), such matching might be better performed probabilistically with a likelihood function assigned to each individual cue (source of landmark information). What does this imply? The Bayesian framework provides a principled approach how to optimally combine different sources of sensory information that might contain uncertainty and how to integrate this sensory information with any prior knowledge the observer may have about the task at hand. Thereby, the uncertain and ambiguous sensory information can be expressed in form of a likelihood function, that contains all available information about e.g., distance and orientation of the landmark to the home position. The likelihood function defined as the probability that a certain pattern of sensory signals (S) is obtained given the state of the world (W): P(S|W). In other words, each source of information does not only provide one single value with absolute certainty, such as a given position in space, but an entire probability distribution indicating the likelihood of each value. Combining different sources of information in this framework is simply achieved by multiplying the different likelihood functions (which if all distributions are Gaussians leads to the well-known weighting scheme (e.g., Ernst and Banks, 2002). Most often it is then assumed that the perceptual response is given by the maximum of the resulting combined likelihood function (the maximum likelihood estimate or MLE). Using the Bayesian framework, we could in principle also include prior knowledge. Prior knowledge P(W) is combine with the likelihood function according the Bayes Theorem, which-in essence -again corresponds to a product of these probability distributions. In this case, it is often assumed that the resulting perceptual response is based on the maximum a posteriori estimate (or MAP estimate). For the purposes of this manuscript, however, we do not consider prior knowledge, which is why we relax to a maximum likelihood estimation (MLE) scheme (for reviews on the application of the Bayesian Framework in cognitive science see e.g., Ernst and Bülthoff, 2004; Cheng et al., 2007; Körding, 2007).

This study addresses the question how different sources of landmark information that contain uncertainty are combined. To this end, we presented participants with a limited set of artificial landmarks in an indoor experimental environment, which allowed us to fully control all configurational cues that are available for homing. The task in these experiments was to return to a previously visited “home” location, which is surrounded by a varying number of either visual or auditory landmarks at different locations. After an initial learning period, the participants were relocated to a previously unknown place and we asked them to return to their starting position. During the outbound travel, we made sure to eliminate any body-based cues that could help them to find back home using path integration. Each single landmark alone did not provide any directional information on their own, because they looked or sounded alike from all directions and the surroundings were uniform in all directions. Between conditions, we changed the ambiguity of the landmark array by manipulating the number and thus the configuration of the available landmarks. In the second experiment, we further tested participants performance in a landmark relocation task. What strategies do humans use to find home when the cues are uncertain and ambiguous? We compared different landmark layouts analyzing participants' homing performance and we evaluated whether humans might use a common integration principle, regardless of the modality (visual or auditory) in which the information was presented. That is, we asked how the navigation performance depended on the number of the landmarks and the modality of presentation. To interpret the behavioral data we applied a probabilistic model based on the maximum likelihood approach (e.g., Ernst and Banks, 2002). From the performance to a single landmark cue we know participants basic homing performance. We therefore asked further whether the MLE model can predict the integration performance based on the single landmark responses without fitting any additional free parameters. In other words, we asked whether humans integrate the available landmark information in a statistically optimal way.

## Materials and methods

Overall 25 participants (17 female) aged between 19 and 30 (mean 23) took part: 10 in the auditory homing study; 15 in the visual homing study of which 5 took part in both visual homing experiments. All participants gave their written informed consent before participating and they were paid 6 € per h. The experiments were approved by the ethics committee of Bielefeld University.

### Conditions and procedure in auditory homing

**Figure 1:**
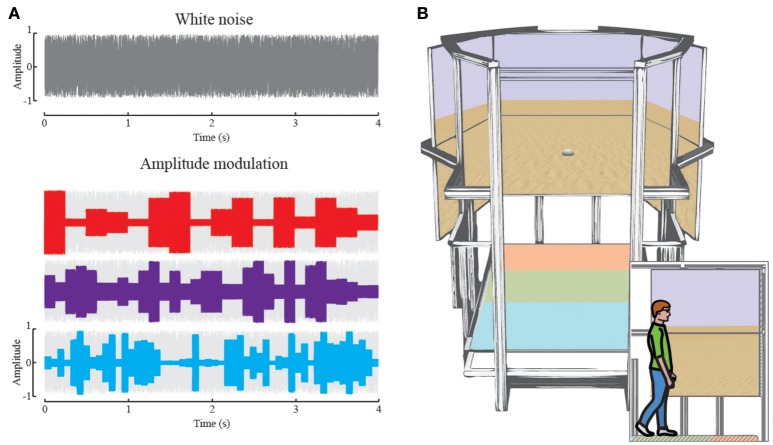
**(A)** Randomly generated white noise (gray). The auditory landmarks played white noise modified by a random amplitude envelope. The envelope changed the amplitude of the white noise randomly after each 250 ms (red, 4Hz), 125 ms (purple, 8Hz) or 76 ms (blue, 13 Hz), respectively. The amplitude ranged from 0.25 to 0.8 where 0 is no sound and 1 is maximum output. **(B)** Rendering the virtual reality setup: In large the setup as seen from the entrance, with 6 high-resolution screens in the back and the treadmill on the bottom. The colored areas on the treadmill correspond to the main functions, acceleration (orange), maintaining speed (green), and deceleration (blue). The screens depict the virtual reality used during this study. The inset depicts a participant walking in the setup, enclosed by the screens for 180° of horizontal and 60° of vertical field of view.

Participants were blindfolded during all auditory homing conditions (Experiment 1-audition) and received no feedback about their performance. These auditory experiments took place in the sports facility (size 27 × 45 m) of Bielefeld University. Throughout the auditory homing task, we used small battery powered speakers (Technaxx MusicMan Mini Soundstation, 50 × 50 × 50 mm) as landmarks. They were placed on the ground pointing upwards such that the sound spread omni-directionally and thus provided the same information independent of the direction of approach. All landmarks were calibrated for equal output volume prior to the experiment. Landmarks were not moved within one experimental block. The landmarks played a white noise sounds with a uniform random amplitude modulation. The gain factor for the amplitude modulation varied randomly with 4 Hz between *g* = 0.25 and 1 (Figure 1A). With this modulation, individual landmarks were distinguishable but not recognizable as such. To aid auditory sound source segmentation, we used an individual landmark sound, which was modulated with a fixed frequency of *f* = 4, 8, and 13 Hz in an additional condition (see Supplement Material). This alternative sound enables participants to identify and learn individual landmarks by the frequency of amplitude changes (Figure 1A). The sounds were played continuously throughout the entire condition.

Prior to the experiment we tested participant's ability to discriminate spatially distributed audio sources. Participants were able to identify only up to three auditory objects that played sounds simultaneously. Consequently, increasing the numbers of landmarks did not improve perception as the landmarks became indistinguishable (see Supplement Material). Based on this result, we decided to use a maximum of 3 landmarks.

**Figure 2:**
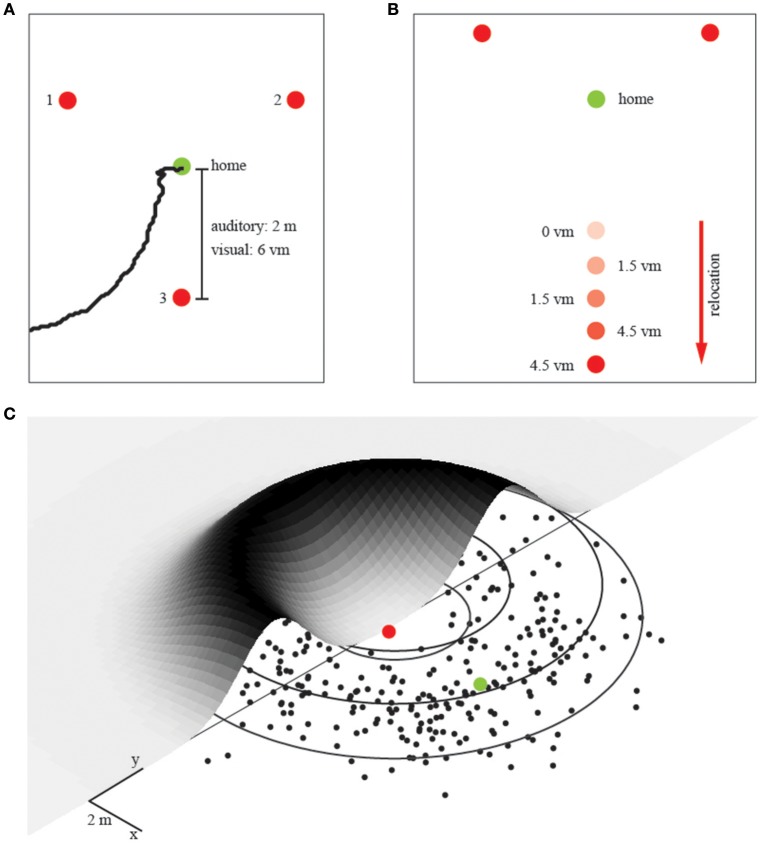
The experimental setup and unimodal prediction. **(A)** Landmark array in the first experiment with increasing number of landmarks. Red dots represent individual landmarks, numbers from 1 to 3 indicate which landmarks were used in the individual conditions (that is, for two landmarks we used landmarks 1 and 2). Distances refer to the auditory (m) and visual (vm) layout. The green dot represents the trained home location. A trajectory recorded with the ceiling camera during the auditory condition is depicted in black **(B)** Landmark array in the second experiment in which we relocated one landmark, again, red dots represent landmark locations. The green dot represents the trained home location. This experiment was only performed in the virtual reality setup and hence done with visual landmarks. We tested overall 5 conditions with relocations in steps of 1.5 vm, as indicated by the corresponding number next to the dot. **(C)** The probability distribution for an ambiguous single landmark. The trajectory endpoints from all participants are collapsed into a single normal distribution, which is then mapped again around the landmark. Single endpoints in black, the ring of equidistance to the landmark in gray. Red dot corresponds to the landmark, green dot to the spatially correct target location during that experimental condition. This probability fit is the basis for all further modeling.

There were three conditions with either 1, 2, or 3 landmarks placed each at a distance of 2 m around the home position (see Figure 2A). Participants learned this home position in an initial training phase which lasted 60 s. That is, participants were placed at the home position when we switched on the individual landmarks. During this training phase participants were not allowed to walk away, but they were able to freely rotate around their body axis. After the training phase, we immediately started the experimental phase. To get participants from this trained home position to the remote start position for the homing experiment (release point), we displaced them passively on a swivel chair. We took randomly chosen, curvy detours to prevent them from getting useful information about the spatial layout of the environment. From the release point they autonomously had to find back to the trained home position. The swivel chair also allowed for quick turns during the detour to further perturb any reliable input from the vestibular organ. In total, there were four different release points. Upon arriving at a release point, participants left the chair and their task was to walk back to the trained position where they thought home was. When they believed to have reached this position, they stopped walking and we recorded their final position with a goniometer with a top-mounted rangefinder, which was set up at the base of the experimental area. This allowed us to later compute the participants end position of each trial with an error of about 5 cm (roughly 0.5% of the entire path walked). We tested 40 trials for each of the three landmark conditions in the audio homing experiments with a break of 10 min between each condition. The sequence of conditions was randomized for each participant.

Subsequently, we run a fourth condition as a control, which was identical to the previous 3-landmark condition except that now we used three auditory landmarks that played individual sounds each. Here we recorded the participants' final positions with a video camera system which had recently been mounted under the Bielefeld University sporting hall ceiling (Gigabit-Ethernet Camera TMC-1327 GE by Jai with a fish-eye lens by Fujinon, 17). The positions were extracted offline and rectified using custom written software (Wilhelm, 2011) resulting in an error between 3 cm in the central visual field of the camera and up to 5 cm in the peripheral area (Figure 2A).

### Conditions and procedure in visual homing

For the visual homing task (Experiment 1-vision), we used a virtual reality setup that consisted of a treadmill platform (custom 2.5 × 1.5 m belt manufactured by Maschinenbau Kitz, Troisdorf, Germany) and six 65” Sony displays arranged in a 3 by 2 layout (Figure 1B) in front of the participant. The participants' feet and heads were tracked by 14 Optitrack Flex 13 cameras (5 cameras aiming on the feet, 9 cameras aiming on the head area). The position of each foot was tracked by three reflective markers. To control the speed of the treadmill for locomotion in virtual reality, the treadmill was divided into three control zones. While walking in Zone 1 (approximately the front 10% of the treadmill length), the treadmill was accelerating until the participant was transported back on the treadmill into Zone 2. While walking in Zone 2 the speed of the treadmill remained constant (approximately the middle 50% of the treadmill length). When the participant slowed down during walking, he/she would be transported further back on the treadmill until they entered Zone 3 of the treadmill in which case also the treadmill slowed down until finally it stopped (approximately last 40% of the treadmill). This way participants could freely adjust their speed of walking and the treadmill would follow their speed. The treadmill allowed only linear forward movements. To control the orientation of visual locomotion, we tracked the position of the participants' head and its orientation with six reflective markers, mounted on a frame worn as glasses. To enable participants to change direction of virtual locomotion, in the virtual environment we used the following control algorithm: When the head was aligned with the treadmill, participants moved straight forward. Deviation from straight-ahead in head orientation was registered by the tracking system and led to rotation of the visual environment in the given direction. To control the directional motion, there was a sensitive angular zone that started at a deviation angle of 22.5° from straight-ahead. That is, there was a central dead-zone where no visual rotation would occur. This was done to allow naturalistic gaze behavior in the frontal body space. The speed of rotation was linearly linked to the heads' deviation from straight-ahead, increasing in speed to a maximum of 45° per second at 90° head rotation. At this speed, it took participants 8 s to do a full turn within the virtual environment, while enabling them to do finer movements within smaller head turn angles. Due to the nature of the linear treadmill and the head-tracking for rotating the virtual environment, participants could not turn actively on the treadmill. That is, participants were always walking forward within the setup, without the possibility to turn around or walk backwards.

The virtual visual scene was an endless desert, which participants could freely walk through. The sky was colored monochrome blue, offering no directional cues. The whole scenery was rendered in real-time using InstantReality software package (Fraunhofer Institute for Computer Graphics Research). We used small white hemispheres on the ground as local visual landmarks. The overall experimental approach was therefore very similar to the auditory homing experiment.

We tested three different conditions in which we provided either 1, 2, or 3 landmarks placed at 6 (virtual) meters around the home position in the virtual environment (Figure 1B). We placed the visual landmarks further apart than the auditory landmarks due to restrictions of the visual field in the virtual setup. Participants learned these positions in a 60 s training phase in the same way as described above for audio homing. That is, they could freely turn around but not walk away. When homing visually, however, participants pressed a button after each trial to be teleported to the next starting position, randomly assigned from a fixed distance of 40 virtual meters to the target location. We tested 40 trials per condition in the visual homing experiments with a break of 10 min between each condition. The order of the conditions was randomly chosen for each participant.

In a second visual homing experiment (Experiment 2), we manipulated the distance between the individual landmarks. While the overall procedure was exactly the same as in the first visual homing experiment, we changed the position of one single landmark when the participant was teleported to a new starting position (Figure 2B). That is, in 5 conditions we relocated one of the three equal looking landmarks between 0 and 6 meters, in steps of 1.5 meters, away in an orthogonal direction to the remaining two landmarks (Figure 1C). The order of these 5 displacement-conditions was randomly chosen for each of the 10 participants.

### Modeling

**Figure 3:**
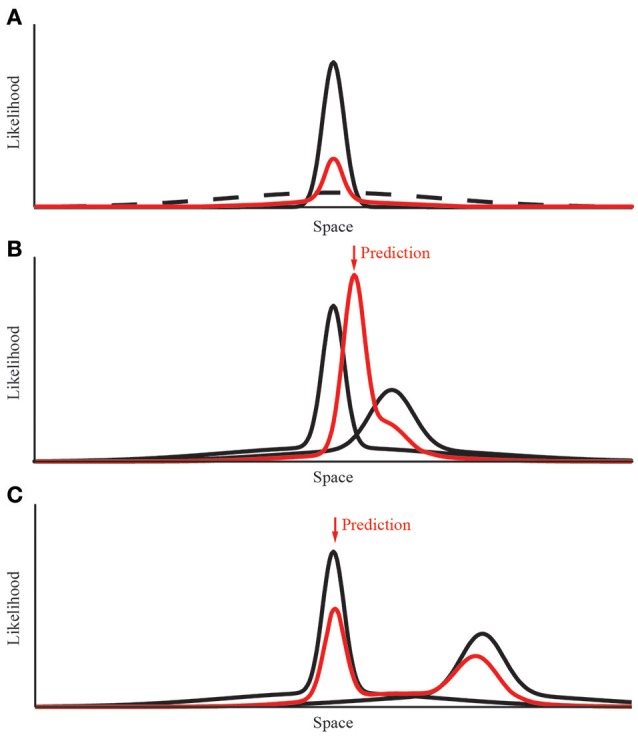
Modeling the integration and breakdown of separate landmark cues. **(A)** Construction of the Gaussian mixture probability distribution. The data derived from the experimental condition is shown in black. We added an additional underlying distribution with 10 times the variance and a relative amplitude of 12%. The resulting distribution is normalized after the summation of both distributions. **(B)** Combining the response probabilities for two different cues. When both distributions overlap, the resulting distribution has a decreased variance (red line). The shift along the x-axis is determined by the quality of both distributions, that is the variance of both cues. **(C)** Modeling the breakdown of cue integration is possible due to the mixture of Gaussian distribution. When the conflict between both cues is too large their respective probability distributions do not overlap. In this case the prediction returns to the single cue with less variance.

We used probabilistic models based on maximum likelihood estimation to predict human homing behavior. We started with a simple “Gaussian Model” with a probability distribution in form of a 2D donut that has a Gaussian shaped ridge (Figure 2). Later, we extended this simple model in order to model the breakdown of integration, replacing the Gaussian ridge by a mixture of two Gaussians (“Mixture Model”). This is because the product of two Gaussians used for modeling the integration will again always result in a Gaussian, such that it is impossible to model the breakdown of integration using simple Gaussians. However, it has been shown previously that with a tiny modification to the shape of the probability distribution—i.e., changing the Gaussian to a Gaussian with heavy tails—this limitation can be overcome (e.g., Knill, 2007; Ernst and Di Luca, 2011). In practice the heavy tails are best modeled by a mixture of two Gaussians where both of them are aligned and where the second Gaussian that is forming the heavy tails has a very large variance (Figure 3; for more details see below).

The information provided by a single (uniform) landmark is the distance to the home position—it is ambiguous in direction. Thus, the likelihood function provided by a single landmark can be modeled as a ring with a radius corresponding to the distance to home. Because there is uncertainty in the distance cue, the likelihood function corresponds to a donut shaped probability distribution as shown in Figure 2B. To quantify participants' homing performance, we fitted such a 2D, donut-shaped probability distribution to the participants' spatial distribution of end-positions. The distribution around one landmark is ring-shaped due to the ambiguity constraints (Figure 2B) and is described by two parameters, the distance to the landmark and the variance of the Gaussian distributed ridge. We obtained both parameters from fitting the model to the data in the single landmark condition: (1) we converted the endpoints into polar coordinates with the landmark in the center, and (2) collapsed the data from all directions onto a single dimension, such that we could fit a Gaussian distribution and determine its variance on the distance axis. We call this the “Gaussian” fit. All further probability distributions for different configurations of landmarks are linear combinations (i.e., multiplications) of this uni-landmark distribution. That is, to model the response probabilities for more than one landmark, we linearly combined the response probabilities of multiple single landmarks by multiplying their distributions. This way we can predict the performance with two and three landmarks without fitting any additional free parameters.

In the second visual experiment, we relocated the landmarks between training and test. Also in this case of a landmark relocation, we used the same parameters as in the first experiment. However, as mentioned above to be able to model the breakdown of integration, we need to extend the Gaussian Model slightly by relaxing the assumption that the donut-shaped probability distribution has a ridge that is Gaussian. Instead, we need to replace this Gaussian assumption by a mixture of two Gaussians, which corresponds to a “Mixture Model” (cf. Knill, 2007; Ernst and Di Luca, 2011; Figure 3). Both Gaussians in the Mixture Model are centered on the home-landmark distance from the landmark. However, they differ in variance: the main information about the distance to the home position provided by the landmark is given by the central Gaussian with a variance coding for the uncertainty with which the home position can be localized. The second Gaussian in the mixture has a much larger variance and essentially acts like a pedestal forming heavy tails (Figure 3). So this model has four parameters: (1) the distance home-target which is the same value as in the previous experiments, (2) the variance of the central Gaussian, which is obtained as before by collapsing the data in the uni-landmark condition, (3) the variance of the Gaussian forming the heavy tails, which is achieved by increasing the variance to cover the experimental space. This way, the heavy tails are essentially flat in the working range that is of interest to the experiment. Increasing the variance of this second Gaussian thus makes only marginal differences. Finally (4), the relative contribution of the central and heavy tail Gaussian which is determined by the relative amplitude of both Gaussians. We fixed this parameter at 12% which provides enough energy in the heavy tails to model the breakdown of integration. The exact number of this parameter is not very critical as long as there is enough energy in the heavy tails while at the same time the central Gaussian remains dominant. Given we wanted to determine all parameters from the One-Landmark Condition allowing us to make parameter-free predictions for all the other conditions, we do not have enough “outlier data” from the homing performance in the periphery of our workspace such that we could fit these two “heavy-tail parameters” reliably. This is why for practicality we simply chose them using a reasonable logic and fixed them henceforth for making predictions. As long as the second Gaussian forming the heavy tails is practically flat in the region of interest—here the workspace—it is sufficient for predicting the breakdown of integration. The modeling is therefore quite robust against the exact choice of variance and amplitude ratio of this second Gaussian as long as it completely covers the navigational space that participants could use. With this minor addition to the traditional MLE approach, the Gaussian Mixture Model is very effective for modeling the breakdown of integration. Such a breakdown of integration has been described before for cues, which are highly conflicting (e.g., Gepshtein et al., 2005).

When different likelihood functions, all made up of the same mixtures of Gaussians, are combined by multiplication, the point of the largest resulting likelihood determines the location that participants should aim for (maximum likelihood estimation). This mixture model can naturally predict the breakdown of integration because of the probability in the heavy tails of the likelihood functions. With small conflicts the center distributions dominate and the product between the likelihood functions corresponds to a unimodal weighted average (cf. Figure 3B). With large conflicts, however, due to the heavy tails the product of the likelihood functions becomes bimodal—i.e., the two distributions remain separate and not integrated (cf. Figure 3C, Knill, 2007; Ernst, 2012).

To emphasize again, all parameters for both the simple “Gaussian” model and the extended “Mixture of Gaussian” model, are derived from the one-landmark conditions. The two- and three-landmark conditions as well as the landmark relocations are parameter-free predictions of the model. That is, for all conditions, the likelihoods derived from the single landmark condition are simply multiplied and the maximum-likelihood estimate is taken as the estimate.

## Results

### Landmark ambiguity

**Figure 4:**
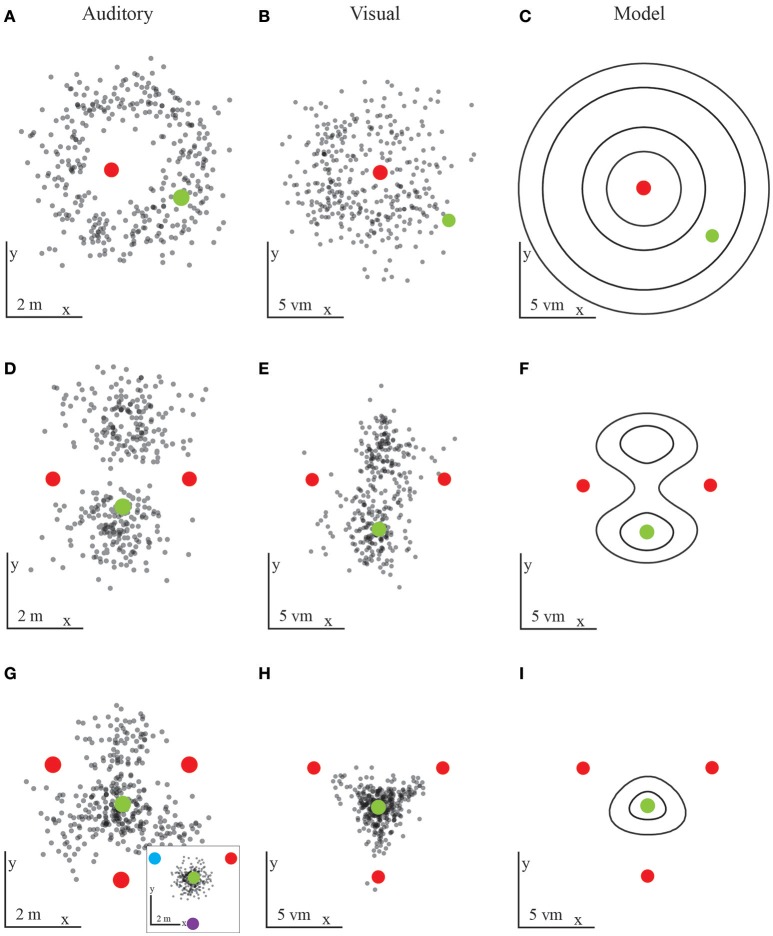
Results from the first experiment with auditory **(A,D,G)** and visual **(B,E,H)** landmarks. Each black dot is the final position of one participants' trajectory, red dots mark landmark locations, and green dots the target location. The predictions **(C,F,I)** are based on the probabilities to reach a certain position. **(A,B)** Results for one landmark in the auditory **(A)** or visual **(B)** condition. The results fall within a ring around the landmark, as predicted by spatial constraints. The probabilistic model is based on the visual condition performance and implies an underlying Gaussian distribution **(C)**. Human participants display large variability in the distance to the landmark. **(D,E)** Results for two landmarks in the auditory **(D)** or visual **(E)** condition. The prediction shows similar regions with similarities to the target locations **(F)**. Here the probabilistic model combines two ambiguous landmarks by combining the rings obtained for one individual landmark. **(G,H)** Results for three equal landmarks in the auditory **(G)** or visual **(H)** condition. In the auditory conditions participants could not identify the target location in all trials. The inlayed figure shows the result for three unique auditory landmarks. When every landmark played a slight modified sound participants' performance increased and is similar to the visual performance. The probabilistic model combines the responses from three individual landmarks in a way closely resembling the human performance **(I)**.

In both, the visual and the auditory experiments we find—maybe unsurprisingly—that participants cannot reliably locate their home position when cues provide ambiguous information. However, as just mentioned above the performance to this baseline condition is needed for fixing all the necessary parameters used for the modeling of the integration behavior of all later conditions. With one omnidirectional uniform landmark, a ring around that landmark represents an infinite number of potentially correct positions with a radius provided by the home-to-landmark distance. This high degree of ambiguity should thus result in responses that fall close to the aforementioned ring around the landmark. This ambiguity constraint is confirmed empirically, both in the auditory and in the visual conditions (Figures 4A–C).

**Figure 5:**
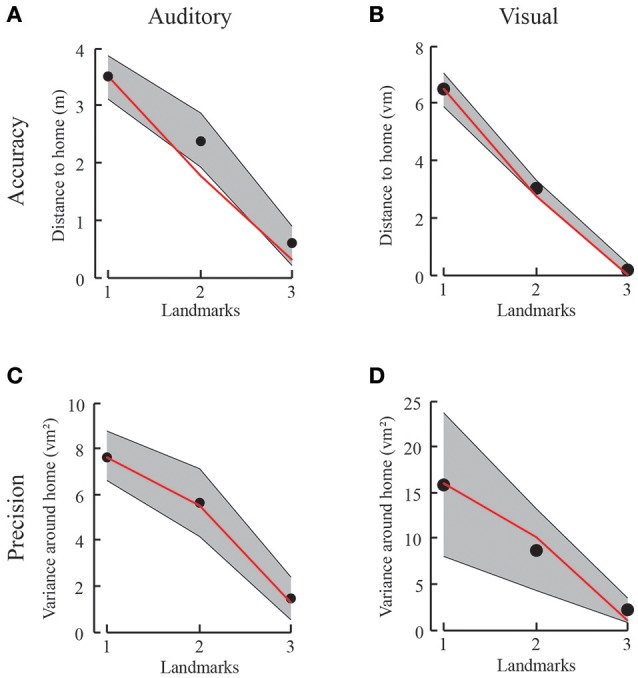
Results from the first experiment with auditory **(A,C)** and visual **(B,D)** landmarks. **(A,B)** Accuracy and model prediction measured from participants' performance in the auditory **(A)** or visual **(B)** condition for each number of landmarks. Black dots depict the mean accuracy for a given condition with gray shaded standard deviation. The red line corresponds to the probabilistic prediction based on the results from only one landmark. **(C,D)** Variance in goal location performance for participants' behavior in the auditory **(C)** or visual **(D)** condition for each number of landmarks in black and model prediction in red. The variance decreases with increasing number of landmarks, just as predicted by the mixture of Gaussian probabilistic model.

When placing a second omnidirectional landmark into the landscape at another location next to the first landmark, we expect the overall ambiguity of the landmark layout to decrease. Because the second landmark is placed at the same distance to the home position as the first one, we expect two ambiguous locations to remain. That is, due to the spatial constraints we predict that the likelihood functions of both landmarks should form a ring of ambiguity around them with a radius defined by the home-landmark distance. The rings formed by the likelihood functions intersect at exactly two positions (unless home is right on the line between the two landmarks, in which case the rings just touch). Thus, the product of the two likelihood rings reduces to a bimodal distribution with its two peaks corresponding to two equally likely positions. These two positions are the learned home position and a second mirror-symmetric position on the other side of the landmarks. Since both landmarks are similar and provide no direction information by themselves, the remaining ambiguity should lead to responses scattered around these two positions. Similarly, the variance of the responses is decreasing when trajectories lead to only two ambiguous locations. This is again true for both the auditory and the visual homing task (Figures 4D–F). With decreasing ambiguity, we thus see increasing accuracy and a decrease in variance. We define accuracy as the distance of all responses from the home location and precision as the spread in the data around the mean. For the Gaussian model this spread corresponds to the standard deviation (i.e., the square root of the variance) of the Gaussian. Therefore, unlike any deterministic model, the probabilistic models used here make clear predictions for both the accuracy and the precision of the homing performance (Figures 5A–D).

We chose a third scenario with three landmarks which by their configuration provide unambiguous information with respect to a home location. That is, we chose three omnidirectional landmarks forming a triangle with the home location in its center. In this unique case, all landmarks are evenly distributed and equidistant. That is, the rings formed by the likelihood functions of each landmark intersect at exactly one position and thus the product of the likelihood functions provides an unambiguous unimodal peak in the center of the three landmarks. For the visual homing task with the three landmarks we observed the predicted behavior: That is, with this layout all participants could accurately and precisely locomote to the learned home position (Figures 4G–I). By contrast, the accuracy for the auditory homing task was predicted less well. Instead, for the auditory condition with three identical landmarks we found a noticeable difference compared to the performance observed in the visual homing task. Therefore, it seems that the configuration of three identical auditory landmarks is not as unambiguous as it was in the visual task. We come to this conclusion because many responses also scatter in-between always two individual landmarks and they are not concentrated in the center of the three landmarks. Finding responses in the areas between two landmarks does not fit the unambiguous geometry of the learned home position given the three landmarks, but rather resembles the data from two landmark experiments.

Why is that? In audition it is harder to segment different sources producing comparable sounds into separate objects. As such, in audition the spatial layout of the scene is much more difficult to perceive. To help auditory segmentation, we next played sounds with a different frequency of amplitude modulation from each of the three landmarks. This way the individual sound sources became identifiable, which added directional information to the landmark array and further reduced ambiguity. The predicted search position, which is based on the geometry of the setup remains the same, i.e. the center of the three landmarks. Given that the three auditory landmarks were now relatively easy to segment, we found that participants could locate the previously learned home location with high reliability and similar accuracy and precision as in the visual homing task (Figure 4G- inlay).

The probabilistic model predicts the probabilities to reach a certain location which coincides well with the participants' behavior. By measuring the performance distribution with respect to only one landmark, we can reliably model the response to alternating numbers of ambiguous cues, both in the endpoints of trajectories (accuracy) and the variance of the data (precision). We find an increase in accuracy (i.e., decrease in the average distance to home) along with the number of landmarks (Figures 5A,B). Similarly, we find a decrease in variance (Figures 5C,D). The one condition which did not follow this pattern of results, i.e., which was different from the model predictions, was the abovementioned three audio landmark condition where all three auditory landmarks were identical. As discussed already earlier the reason for this deviance can be found in the difficulty to segment audio sound sources that are identical.

We test individually for all participants and both auditory and visual conditions whether the distance to the home location is decreasing with increasing landmark number. We compare the regression slope across the three conditions to the alternative hypothesis of having no trend [student's *t*-test, *t*_(9)_ = −31, *p* < 0.001, slopes between −3.48 and −2.47 when fitting a linear regression model (Figure 5)]. We find a significant increase in accuracy, that is, the distance between the correct home location and the end of each trajectory is decreasing. This is also the case for the decrease in variance [student's *t*-test, *t*_(9)_ = −4, *p* < 0.01, for all participants, slopes between −13.32 and −3.51]. Comparing accuracy and variance between experimental data and the “Gaussian Model” predictions show no significant difference for any landmark combination. Here we tested prediction and data for each condition: One-tailed paired-sample *t*-test, values between *t*_(9)_ = 0.37, *p* = 0.71 and *t*_(9)_ = −1.75, *p* = 0.25 for accuracy. One-tailed paired-sample *t*-test, values between *t*_(9)_ = −0.71, *p* = 0.75 and *t*_(9)_ = 0.92, *p* = 0.19 for variance. Power analyses with the given effects indicate that we would need over 100 participants to reach a significance level of 0.95 when comparing the behavioral accuracy with the prediction from the model, and over 2,000 participants for comparing behavioral and the model predicted precision. These analyses together support our claim of a non-significant difference between the model and the empirical data. Therefore, we conclude that the information from three landmarks is combined using individual landmark cues in a fashion indistinguishable from optimal considering that the decision is based on the maximum likelihood estimate. Since none of the deterministic models that e.g., rely on distance or direction information makes any prediction for the precision of the homing performance, it here makes no real sense to perform any model comparison with these models. Therefore, we decided to omit such a comparison.

In the following experimental condition, we need to extend the simple “Gaussian” model to capture the breakdown of integration. This is done using the Mixture Model described above. To verify that this extended model does not change the results of these 1, 2, and 3-Landmark Conditions, we also tested the prediction of the extended Mixture Model against our results and found no significant difference when comparing the two model predictions [two-tailed paired-sample *t*-test *t*_(2)_ = 2, *p* = 0.18 for accuracy and *t*_(2)_ = 0.31 *p* = 0.78 for variance in all landmark conditions]. That is, for this “Number of Landmark” Experiment the additional parameters of the extended Mixture Model do not make a difference.

### Conflicts in landmark reliability

**Figure 6:**
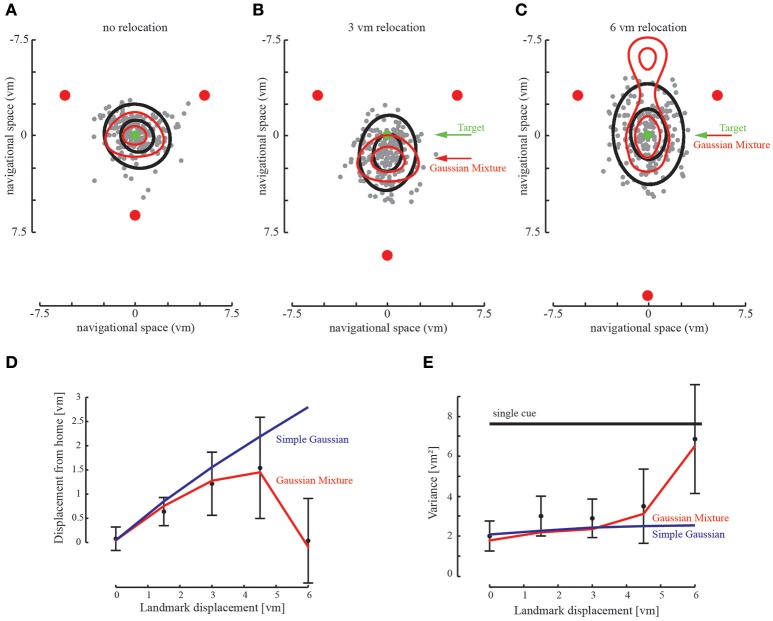
Relocation of landmarks creates a conflict between the landmark cues. **(A–C)** Participants' responses shift with the relocated landmark. The prediction (red ellipse) fits well to the behavioral data of all subjects (black ellipse containing 95% of responses). Gray dots indicate the endpoints of all participants in the given experimental condition. Green arrow points to the trained home location, red arrow points to the maximum of the probabilistic prediction. **(C)** When the conflict is too large, participants return to the proximal landmarks. However, we find that participant endpoints are only situated at one side of the landmark array. The extended Gaussian Mixture model also predicts a return to the two stationary landmarks. At the given landmark relocation we find that the location with the largest probability is similar to the home location. Only with further landmark relocation we would find full ambiguity in the model (not shown). **(D)** Accuracy as determined by the distance to the target location. Accuracy decreases with landmark relocation, as predicted by the Gaussian Mixture model. When the landmark is repositioned by 6 vm accuracy returns to the initial value. The Simple Gaussian model used in the first experiment predicts the integration during the relocation, but not its breakdown. **(E)** Variability defined as the area covered by the standard deviation of the behavioral data. Variability decreases when cues are optimally integrated and is smaller than the variability of only two combined landmarks. When the landmark is relocated by 6 vm variability increases and reaches single cue level. The Simple Gaussian model predicts integration and no increase in variability.

To further examine how spatial information from different landmarks is combined, we introduced changes in the visual landmark array between training of the home position (i.e., remembering the landmark configuration relative to home) and the respective test to find back home. Particularly, we were interested in how these changes affect the homing performance as this reveals how the structure of landmarks is integrated into a whole for navigation. To this end, we relocated one landmark outwards between training and test (Figures 6A,B). These changes were introduced very subtly and thus went unnoticed by the participant, which we confirmed by debriefing the participants after the experiment. We found that with small changes participants average homing performance is affected by the relocated landmark. That is, their locomotion end points are not centered on the learned home position but pulled toward the relocated landmark, which marks the new center of the rearranged three landmarks. Thus, this behavior with small relocations confirms that the landmark information is integrated into a whole spatial configuration with the new home position defined by the maximum likelihood position in its center.

Probably even more interesting, this behavior is reverted when the third landmark is relocated more than 5 virtual meters between training and test. With such large changes, we observed that participants reverted to use the two landmarks which remained at the initial location, ignoring the third deviant landmark (Figures 6C,D). This is a clear sign of break-down of integration of the spatial layout information and a vetoing of the third deviant landmark. To model these results, we used the same probabilistic approach described earlier based on maximum likelihood estimation, but this time using the mixture of Gaussians as the likelihood function (Mixture Model) instead of the simple Gaussian Model. The goal was again to predict participants' behavior in the three landmarks condition based on empirical data from the homing behavior in the one landmark condition of the previous experiment without any additional free parameters.

When the third landmark is relocated by a small amount the Mixture Model predicts that the homing behavior of participants follows the deviant landmark and as such according to this model the homing performance should be determined by the new center of the landmark configuration after relocation. With small relocations, when the central Gaussians of the individual probability distributions still largely overlap, we find that the empirical data follow this prediction very closely.

When the third landmark is further relocated (beyond 5 virtual meters), the conflict between training and test of the available landmark cues is increased. According to the model, with larger relocations the overlap between the center Gaussians of the likelihood functions of the deviating landmark and the other two decreases (cf. Figure 3B). Instead the heavy tails of the mixture of Gaussians will gain more influence and the product between the likelihood functions will become bimodal (Figure 3C). As a result, we find that the Mixture Model predicts a return to the two adjacent landmarks and not to the single third landmark (Figure 6C). But the two possible locations of the remaining two landmarks are not exactly equally likely as they have been in the Two-Landmark Condition. In contrast, the location on the side of the third landmark is emphasized by the prediction reaching a higher probability, because the heavy tails have an asymmetric influence on both sides of the two unchanged landmarks (Figure 6C). This prediction coincides very well with human behavior. The experimental data indicate that the two unchanged landmarks are not ambiguous when a third landmark is available, even when the conflict, that is, the relocation is large. Even when integration of the third landmark breaks down it is still used for disambiguation of the remaining landmarks.

To compare the model prediction to the empirical homing locations, we compute the difference between the prediction and the average participants' data. We repeat this for accuracy (the distance to the correct home location) as well as precision (the variance of the data, measured by the area of the ellipsoid containing 95% of the participant responses as depicted in Figure 6, or measured by the area of the prediction, covering 95% of the probability). This reveals no significant differences in accuracy [paired-sample *t*-test, *t*_(4)_ = 1.16, *p* = 0.31 on the hypothesis that the difference between model and prediction is 0] and variance [paired-sample *t*-test *t*_(4)_ = 2.36, *p* = 0.07 on the hypothesis that the difference between model and prediction is 0]. Thus, the Mixture Model seems a good description of the human behavioral data.

By contrast, when we model the same relocation data using the simple Gaussian Model, we find a significant difference to the participants' data both in accuracy [paired sample *t*-test *t*_(4)_ = −2.85, *p* < 0.05 on the hypothesis that the difference between model and prediction is 0] and variance [paired-sample *t*-test *t*_(4)_ = 4.43, *p* < 0.01 on the hypothesis that the difference between model and prediction is 0]. This clearly indicates that by contrast to the Mixture Model, the simple Gaussian Model cannot explain the human behavioral data and can thus be rejected. Again, since none of the deterministic models make any prediction for the precision of the homing performance, we decided to also omit such a model comparison here.

In conclusion, when integration breaks down the more reliable landmarks are preferred and the performance returns to the target location, resulting in increased accuracy and variance, as also predicted by the Mixture Model. This suggests that precision is sacrificed for accuracy (Figure 6E). Only when the disambiguating landmark is relocated far enough we expect a return to the two landmark cue probabilities, as the third landmark would not be considered as relevant anymore.

## Discussion

We investigated human homing behavior with different configurations of auditory and visual landmarks. We found that both types of landmarks are used in a similar way for homing. This suggests that the underlying navigational mechanism might be general and not specific for each sensory modality. Such hints at common mechanisms have been discussed for time, space, and quantity (e.g., Walsh, 2003) and many empirical studies find that cue combination works similar for different modalities (e.g., Trommershauser et al., 2011 for an overview).

### Sensory processing for directional hearing and seeing

How does the navigation performance depend on the number and type of landmarks? Overall, we found that in the auditory and visual homing tasks, the number of landmarks and thus the degree of ambiguity affects both the accuracy and the precision of human homing behavior. With an increasing number of landmarks, the spatial ambiguity is reduced and the participants seem to be taking advantage of this. However, at first glance there seemed to be one exception: the behavior in the auditory task with three identical landmarks showed that the ambiguity is higher compared to the visual task with three landmarks. As reason we have identified the segmentation problem in audition with identical sound sources, which again introduced spatial ambiguity. Making the sound source identifiable helped solving the segmentation problem and as such reduced ambiguity. In turn, presenting the sources which were individually identifiable increased precision and accuracy in the auditory homing task with three landmarks to a level comparable to visual homing with three landmarks. It might seem surprising that such a seemingly small change in the sound profiles of the auditory landmarks could improve the results to such a degree that it became comparable to the homing performance observed with visual landmarks. A likely reason might have been the differences in the acquisition and processing of spatial information between vision and audition (Alain et al., 2001, see Bregman, 1994 for a synopsis): Vision is spatiotopically organized and might allow for an easy segmentation of non-occluded objects in the visual field. As such, in general humans can discriminate spatially distributed visual landmarks even if they are identical in their appearance. On the contrary, audition is organized in frequency maps and one has to infer spatial location of the sound sources from the difference in sound profiles between the two ears. If two sound sources emit similar frequency content it becomes very difficult for the auditory system to divide the sound sources into individual objects. Instead individual objects with sounds that are very similar might be perceptually merged. Think of a pair of speakers playing two exact same sound signals: the perceived sound is located right in between both speakers. Solving this auditory ambiguity problem can be achieved by separating cues not only by location, but also by the characteristics of the sound profiles. Another difference between the two modalities is that auditory cues can be heard all the time, whereas visual objects can only be updated when they are in the visual field. Interestingly, our preliminary test revealed that participants were able to correctly count at least 3 auditory landmarks with identifiable, yet similar sounds. During the experimental testing, participants were unable to use this knowledge to locate the target location until we separated the cues also by the characteristics of the sound profiles. In case of multiple cues participants could benefit from redundant information, when cues differ not only in their physical position but also in the profile of the sound envelope.

### Models for landmark navigation

We find that participants integrate the available information in a near to optimal fashion in terms of our probabilistic model, by reducing the variability of homing behavior. The behavioral data are very similar to the parameter-free predictions of the probabilistic models, which provide the benchmark for optimal performance in form of an ideal observer. That is, integration of an increasing number of landmarks is well predicted by the Gaussian Model, which is based on multiplying the individual likelihood functions as derived from the single landmark condition. Optimal integration also occurs when one landmark is relocated. However, it breaks down in favor of higher accuracy but less precision when the third landmark is displaced too far, i.e., when the conflict between the learned and the tested configuration becomes too large (Figures 6A–C). However, this breakdown is not described by the simple Gaussian Model. Both, integration of numerous landmarks and also its breakdown is well predicted by the probabilistic MLE integration model assuming a mixture of two Gaussians as the likelihood function (Figures 6D,E). This model parsimoniously solves the problem that arises at larger conflicts between signals where, in order to behave robustly, integration should break down (cf. Knill, 2007; Körding et al., 2007; a discussion of the slightly different models for the breakdown of integration can be found in Ernst and Di Luca, 2011 and in Ernst, 2012).

Many psychophysical studies provide evidence for optimal cue integration in human multisensory perception (e.g., review: Ernst, 2006). The situation is less clear in the context of navigation, even though there is growing evidence that navigating animals also combine cues based on Bayesian principles (Cheng et al., 2007). Studies on ant navigation suggest that different systems are in operation simultaneously integrating different sources of information (Collett, 2012; Legge et al., 2014; Wystrach et al., 2015; Fleischmann et al., 2016) and evidence from landmark navigation studies in pigeons (Sutton, 2002; Blaisdell and Cook, 2005; Cheng et al., 2006) or cue integration in monkeys (Fetsch et al., 2010; Dokka et al., 2015) also indicates close to optimal cue integration.

Still, when it comes to modeling navigation behavior it is often suggested that one navigational system, such as path-integration or snapshot matching are solely used for certain tasks and places during a journey. That is, they are not combined but one system is dominating the behavior at a time (Etienne et al., 1996). In consequence, there are several homing models that focus on “view-based” methods only. In these models an image, which is not necessarily a static snapshot (Cartwright and Collett, 1983, 1987; Collett and Rees, 1997; Zeil et al., 2003; Möller and Vardy, 2006; Graham and Cheng, 2009; Basten and Mallot, 2010; Dittmar, 2011), is taken at a certain position and stored in memory. During homing, this snapshot is constantly compared to the current view of the scenery (e.g., Stürzl et al., 2016). This approach can also be used in autonomous agents, which have the computing power to do pixel by pixel comparisons (Zeil et al., 2003). These models often rely on geometric constraints, such as angles or distances between landmarks. Waller et al. (2000) tested whether humans use a combination of distance and bearing information to relocate to a target location. Consistent with our data from Experiment 1, he could show that participants mainly rely on distance information to the landmarks. It is noteworthy that one of the participants tested in Experiment 1, displays a behavior in the three visible landmark condition which would fit to the bearing hypothesis by Waller, leading to response shapes pointing to individual landmarks. This is not the case for any of that other 15 participants tested in this Experiment 1. Using the distance and bearing approach by Waller et al. (2000), however, is not enough to predict the breakdown of integration observed in Experiment 2, as it does not predict how to weigh information in case of discrepant information. A different approach from robotics navigation is the average landmark vector model (Lambrinos et al., 1998), which requires geocentric knowledge about the current position, rendering the model immune to landmark ambiguities.

In contrast, our model is based on empirical data, making it possible to determine the probability for localizing a given spatial position with a varying number of landmarks. We do not know how participants identify individual landmarks and for our model this is irrelevant since the only input is the empirical homing performance with one landmark. It is a model for cue combination only and the detailed analysis of characteristic homing errors allowed us to elucidate how humans combine multiple landmark cues and how to model this integration behavior. By contrast a recent study focuses on reconstruction-based hypotheses for a scene-matching, showing that it is possible to use scene information to reconstruct the spatial layout of a navigation setup (Pickup et al., 2013). A comparison between empirical homing data and likelihood maps generated by their homing algorithm, allows the authors to quantify the success of different reconstruction-based models. As in the present study, error distributions in Pickup et al. (2013) varied substantially with changes in scene layout, which is very similar to our study, but Pickup et al. did not vary the number of available landmarks or changed the spatial landmark setup between training and test as we did here. Interestingly, simultaneous localization and mapping (SLAM) algorithms from robotics that involve probabilistic approaches for cue combination, suffer from similar constraints as human navigation performance when the objects to be recognized are ambiguous (Cummins and Newman, 2008; Mullane et al., 2011).

Even though initial models exist on how to implement Bayesian optimal cue integration in population codes (Ma et al., 2006), we do not want to go as far as speculating how such navigational integration behavior might be implemented neuronally in the human brain (Krakauer et al., 2017).

### Cue integration and breakdown

Maximum Likelihood Estimates have been widely used to explain behavioral phenomena, as in visual-audio or visual-haptic perception (e.g., Ernst and Banks, 2002; Alais and Burr, 2004). It has also been applied to human locomotion behavior (Butler et al., 2010). Thus, it seems reasonable that this mechanism could be used for navigation as well. This is supported by findings of Nardini et al. (2008), who showed that optimal landmark cue integration develops with age, such that children below age 10 behave suboptimally. The probabilistic model determines the certainty of using a single landmark cue (i.e., the relevant information the landmark provides with respect to the target location—here: home) and assumes that all further combinations of the same cue are based on multiplications of the single-landmark likelihood distributions. Our Gaussian mixture model is determined by only four parameters: (a) The distance estimate of the target to the directionally ambiguous landmark, (b) the variance of this estimate, (c) the variance of the second Gaussian distribution and (d) the ratio between the two underlying Gaussian distributions (cf methods: modeling). It does not only fit the empirical data for one cue, but also for other combinations, describing the spatial position, as well as its variability.

Another feature of cue integration is that it eventually breaks down, such that observers rely on single-cue estimates instead of the integrated estimate when information between cues becomes largely discrepant. The probabilistic model described here can predict this breakdown by modeling the likelihood provided by each landmark as a mixture of two Gaussian distributions—one with a variance corresponding to the uncertainty in the target-landmark distance estimate and the other one with a very large variance acting as a pedestal, both centered at the target-landmark distance around the landmark.

Quite interestingly, the probabilistic approach we describe here requires an estimate about the quality of information from each landmark. That is, it requires an estimate of the shape of the probability distribution (the variances of the Gaussians and their relative contributions). As experimenters, we can estimate the distribution by fitting the Gaussian distribution to our single landmark data and then predict the performance using multiple landmarks. How the participants acquire these estimates about the landmark uncertainty while navigating the natural world remains unclear. However, there is quite some evidence that participants are able to acquire this information online while performing cue integration the tasks (e.g., Ernst and Banks, 2002).

We assume that cue integration and its breakdown are important in natural navigation, where landmarks could be potentially relocated, since other people might have manipulated the environment, while we were away. Using probabilistic models in such cases seems reasonable since we can determine with some probability whether a particular landmark has changed. Such probabilistic models have been applied very successfully in other domains investigating human multisensory perception and integration behavior (Ernst and Banks, 2002; Alais and Burr, 2004; Butler et al., 2010; Fetsch et al., 2010). In this experiment, the participants seem to be using the third landmark for disambiguation, increasing the accuracy, thus balancing precision and accuracy in for homing. This is an optimal solution for such a homing task, in which the goal is to reach the target as accurately and as precisely as possible.

Even though landmark ambiguity may be much less of a problem in a natural scene due to the fact that natural landmark information is less redundant and repetitive than the information provided in our experiment. This reduced cue experiment serves as a proof that humans use the available information in a probabilistic fashion and making optimal decisions about the use of this information for navigation. In a similar way probabilistic information may be used in more natural tasks for example when a potential landmark object occurs too often it most likely becomes negligible as a cue for navigation. As an example consider a tree which is a reliable landmark when encountered as a single object in the desert. However, when you encounter the same tree in the forest, probabilistically it will provide less useful information as a landmark for navigation. Taken together, using such redundant landmarks for empirical tests helps us to determine the underlying mechanisms, which are otherwise hard to uncover. It also seems likely that humans use redundant strategies, such as landmark navigation in combination with path-integration, and homing algorithms to aid navigation in different environments, in which reliable landmarks can be occluded by other objects or where they are sparsely distributed.

## Ethics statement

This study was carried out in accordance with the recommendations of the American Psychological Association (APA) with written informed consent from all subjects. All subjects gave written informed consent in accordance with the Declaration of Helsinki. The protocol was approved by the ethics committee of Bielefeld University.

## Author contributions

SJ, ME, and NB designed research. JF and ME designed and built the setup. SJ performed behavioral research. SJ and NB analyzed the data. SJ, ME, and NB wrote the paper. All authors contributed with comments and suggestions to the improvement of the manuscript

### Conflict of interest statement

The authors declare that the research was conducted in the absence of any commercial or financial relationships that could be construed as a potential conflict of interest.
